# Supplementary material for: The Childhood Opportunity Index 2.0: Factor Structure in 9–10 Year Olds in the Adolescent Brain Cognitive Development Study
Source: Int J Environ Res Public Health. 2025 Feb 6;22(2):228. doi: 10.3390/ijerph22020228 (PMC11855348; doi:10.3390/ijerph22020228)
Supplement: Supplementary file 1 [file ijerph-22-00228-s001.zip › ijerph-3344221-supplementary.pdf]

Measures

Individual socioeconomic status.

Household income was originally coded into 10 ordered categories, which then were clustered into tertiles to account for skewness. These categories include less than 50K, between 50 and 100K, and greater than 100K. Parental education was measured by self-reported highest level of educational attainment from both parents. Parent education was then calculated using the average level of education of both parents in years. Highest parental education of one parent was used when data for both parents was missing or a single-parent household. Responses were coded into quintiles including less than a high school education, completed high school/GED, completed some college, and completed a Bachelor's degree.

Supplemental Results

Stand-alone indicators and external criterion.

AP Enrollment, Child Education Centers, Commute Duration, Hazardous Waste Sites, Industrial Pollutants, PM2.5, and Teacher Experience were not significantly associated with any of the outcomes after FDR adjustments.

Employment Rate. Rate of employment was significantly negatively associated with BAS Drive (Standardized B = -0.067, pFDR < 0.001), BAS Fun-Seeking (B = -0.047, p = 0.003) and BAS Reward Responsiveness (B = -0.045, pFDR = 0.003). Rate of employment was significantly positively associated with internalizing symptoms (B = 0.052, pFDR = 0.040). Employment rate was not significantly associated with BIS scores, UPPS-P scores, or ASEBA CBCL Externalizing, Thought or Attention Problems.

High School Graduation Rate. High School Graduation Rate was significantly negatively associated with BAS Drive (Standardized B = -0.036; pFDR = 0.035) and BAS Reward

Responsiveness (B = -0.035; *p*FDR = 0.041). High School Graduation Rate was not significantly associated with the BIS, UPPS-P Sensation Seeking, Negative Urgency, Lack of Planning, or Lack of Perseverance. High School Graduation Rate was not significantly associated with the CBCL Internalizing, Externalizing, Attention Problems, or Thought Problems.

School Poverty. School Poverty was significantly positively associated with BAS Drive (B = 0.064; *p*FDR < 0.001), BAS Fun-Seeking (B = 0.041; *p*FDR = 0.034), BAS Reward Responsiveness (B = 0.045; *p*FDR = 0.018), and significantly positively associated with UPPS-P Lack of Planning (B = -0.044, *p*FDR = 0.017). School Poverty was not significantly associated with ASEBA CBCL subscales, BIS scores, or UPPS-P Sensation Seeking, Negative Urgency, Positive Urgency or Lack of Perseverance.

Home Ownership. Home Ownership was significantly negatively associated with BAS Drive (B = -0.045, *p*FDR = 0.003) and CBCL Thought Problems (B = 0.035, *p*FDR = 0.046). Home ownership was not significantly associated with the BIS, UPPS-P, or BAS Fun-Seeking and BAS Reward Responsiveness. Additionally, it was not associated with the Attention Problems, Internalizing, and Externalizing ASEBA CBCL subscales.

Heat Exposure. Heat Exposure was significantly positively associated with BIS scores (B = 0.052, *p*FDR = 0.032). Heat Exposure was not significantly associated with BAS subscales, UPPS-P subscales, or ASEBA CBCL subscales.

Ozone. Ozone was significantly negatively associated with UPPS-P Lack of Planning (B = -0.034, *p*FDR = 0.019). Ozone was not significantly associated with any of the ASEBA CBCL, BIS, BAS, or the UPPS-P Positive Urgency, Negative Urgency, Sensation-Seeking, or Lack of Perseverance.

Table S1. Eight Factor Solution of the Childhood Opportunity Index 2.0

Indicator	Factor 1	Factor 2	Factor 3	Factor 4	Factor 5	Factor 6	Factor 7	Factor 8
AP Enrollment	0.21	0.16	0.31	0.15	-0.10	0.05	0.08	-0.08
Child Education Enrollment	0.29	0.15	0.08	-0.01	-0.04	0.07	0.13	0.03
CEC	0.06	0.95	-0.05	0.00	0.01	0.00	0.08	0.04
College Enrollment	0.40	0.12	-0.02	-0.18	-0.34	0.15	-0.12	0.13
Commute Duration	-0.29	0.09	0.02	-0.14	-0.22	0.22	0.35	-0.10
Education Attainment	0.86	0.07	0.12	-0.02	-0.03	-0.07	0.02	-0.02
Employment Rate	0.07	0.07	0.03	-0.03	0.01	-0.76	-0.16	0.18
Gray Space	-0.13	0.66	-0.02	-0.12	0.12	0.02	-0.17	-0.25
Hazardous Waste	0.01	0.05	0.01	0.00	-0.01	-0.02	-0.04	0.01
Health Insurance	0.49	-0.23	0.00	-0.25	0.15	-0.12	0.12	0.17
Healthy Food	0.09	-0.07	-0.01	-0.03	0.13	0.64	-0.08	0.18
Heat Exposure	0.01	-0.04	0.04	0.94	0.01	-0.01	-0.01	-0.00
High Skill Employment	0.83	0.04	0.09	0.02	-0.02	-0.10	0.06	0.02
Homeownership	-0.08	-0.26	0.05	0.02	0.08	-0.37	0.48	0.19
Household Income	0.38	0.06	0.16	-0.05	0.01	-0.11	0.5	-0.07
Housing Vacancy	0.08	-0.02	-0.17	0.19	0.09	0.48	-0.06	0.14
High Quality CEC	0.14	0.69	0.01	0.01	-0.00	-0.00	0.04	0.18
HS Graduation Rate	0.09	-0.25	0.17	-0.25	0.15	0.00	-0.05	-0.08
Industrial Pollutants	-0.01	0.06	-0.01	-0.01	0.92	0.039	-0.00	0.015
Ozone	0.04	-0.08	-0.17	0.09	0.03	-0.11	0.04	-0.45
PM2.5	-0.04	0.31	-0.04	0.13	0.36	0.06	0.03	-0.15
Poverty Rate	-0.02	0.04	-0.09	0.02	-0.03	0.77	-0.18	-0.08
Public Assistance	-0.26	0.09	-0.03	-0.04	-0.00	0.72	-0.02	0.17
School Poverty	-0.35	0.30	-0.41	0.19	-0.09	0.07	-0.08	0.00
Singe-Family Household	-0.20	0.13	-0.15	0.04	0.03	0.46	-0.201	0.19
Teacher Experience	0.20	-0.10	-0.34	0.19	0.18	0.22	0.02	-0.08
3rd Grd. Math Prof.	-0.01	0.00	0.99	0.00	-0.01	0.00	0.00	0.01
3rd Grd. Reading Prof.	0.02	-0.00	0.97	0.04	0.00	0.00	-0.02	0.01
Walkability	-0.02	0.63	0.03	-0.17	0.12	-0.01	-0.19	-0.13

*Indicates a correlation greater than 0.40 at $p < .05$; CEC= Child Education Centers; HS = High School

Table S2. Two Factor Solution of the Childhood Opportunity Index 2.0

Indicator	Factor 1	Factor 2
AP Enrollment	0.39	0.12
Child Education Enrollment	0.37	0.20
CEC	0.04	0.82
College Enrollment	0.31	0.33
Commute Duration	-0.23	-0.04
Education Attainment	0.99	0.29
Employment Rate	0.56	-0.11
Gray Space	-0.23	0.67
Hazardous Waste	0.02	0.06
Health Insurance	0.69	-0.08
Healthy Food	-0.35	0.19
Heat Exposure	-0.22	-0.23
High Skill Employment	0.98	0.23
Homeownership	0.43	-0.56
Household Income	0.82	-0.06
Housing Vacancy	-0.44	0.16
High Quality CEC	0.18	0.64
HS Graduation Rate	0.25	-0.13
Industrial Pollutants	-0.07	0.13
Ozone	-0.06	-0.06
PM2.5	-0.17	0.30
Poverty Rate	-0.68	0.33
Public Assistance	-0.74	0.22
School Poverty	-0.78	0.22
Singe Family Household	-0.70	0.27
Teacher Experience	-0.26	0.06
3rd Grd. Math Prof.	0.70	-0.13
3rd Grd. Reading Prof.	0.70	-0.12
Walkability	-0.04	0.69

*Indicates a correlation greater than 0.40 or less than -0.40 at $p < .05$

CEC= Child Education Centers; HS = High School

Table S3. Four Factor Solution of the Childhood Opportunity Index 2.0

Indicator	Child Education	Neighborhood Poverty	Neighborhood Engagement	Socioeconomic Attainment
AP Enrollment	0.30	0.02	0.10	0.19
Education Attainment	0.10	-0.07	0.07	0.88
College Enrollment	0.08	0.33	0.07	0.48
Child Education Enrollment	0.07	0.04	0.09	0.34
HS Graduation Rate	0.17	-0.06	-0.12	0.07
3rd Grd. Math Prof.	1.00	0.00	0.00	-0.01
3rd Grd. Reading Prof.	0.94	0.00	-0.01	0.03
School Poverty	-0.40	0.15	0.24	-0.36
Teacher Experience	-0.39	0.18	-0.10	0.14
CEC	-0.07	0.03	0.84	0.18
High Quality CEC	-0.01	0.08	0.56	0.28
Access to Healthy Food	-0.02	0.73	-0.14	0.15
Green Space	-0.02	-0.03	0.86	-0.17
Heat Exposure	-0.08	0.06	-0.26	-0.13
Health Insurance	-0.02	-0.16	-0.23	0.58
Ozone	-0.15	-0.29	0.07	-0.14
PM2.5	-0.12	-0.04	0.36	-0.08
Housing Vacancy	-0.20	0.57	-0.11	0.10
Walkability	0.04	-0.01	0.81	-0.01
Hazardous Waste	0.01	0.01	0.05	0.02
Industrial Pollutants	-0.20	-0.11	0.16	0.01
Poverty Rate	-0.04	0.83	0.08	-0.07
Public Assistance	-0.01	0.78	0.02	-0.20
Homeownership	0.00	-0.54	-0.43	0.02
High Skill Employment	0.06	-0.10	0.01	0.87
Median Household Income	0.14	-0.39	-0.04	0.44
Employment Rate	0.00	-0.62	0.06	0.14
Commute Duration	0.10	0.06	0.04	-0.26
Single Family Households	-0.14	0.64	0.08	-0.14

*Indicates a correlation greater than 0.40 or less than -0.40 at $p < .05$

CEC= Child Education Centers; HS = High School

Figure S1 Visual Representation of Linear Mixed Effects Regressions of Stand-Alone Indicators and External Criterion

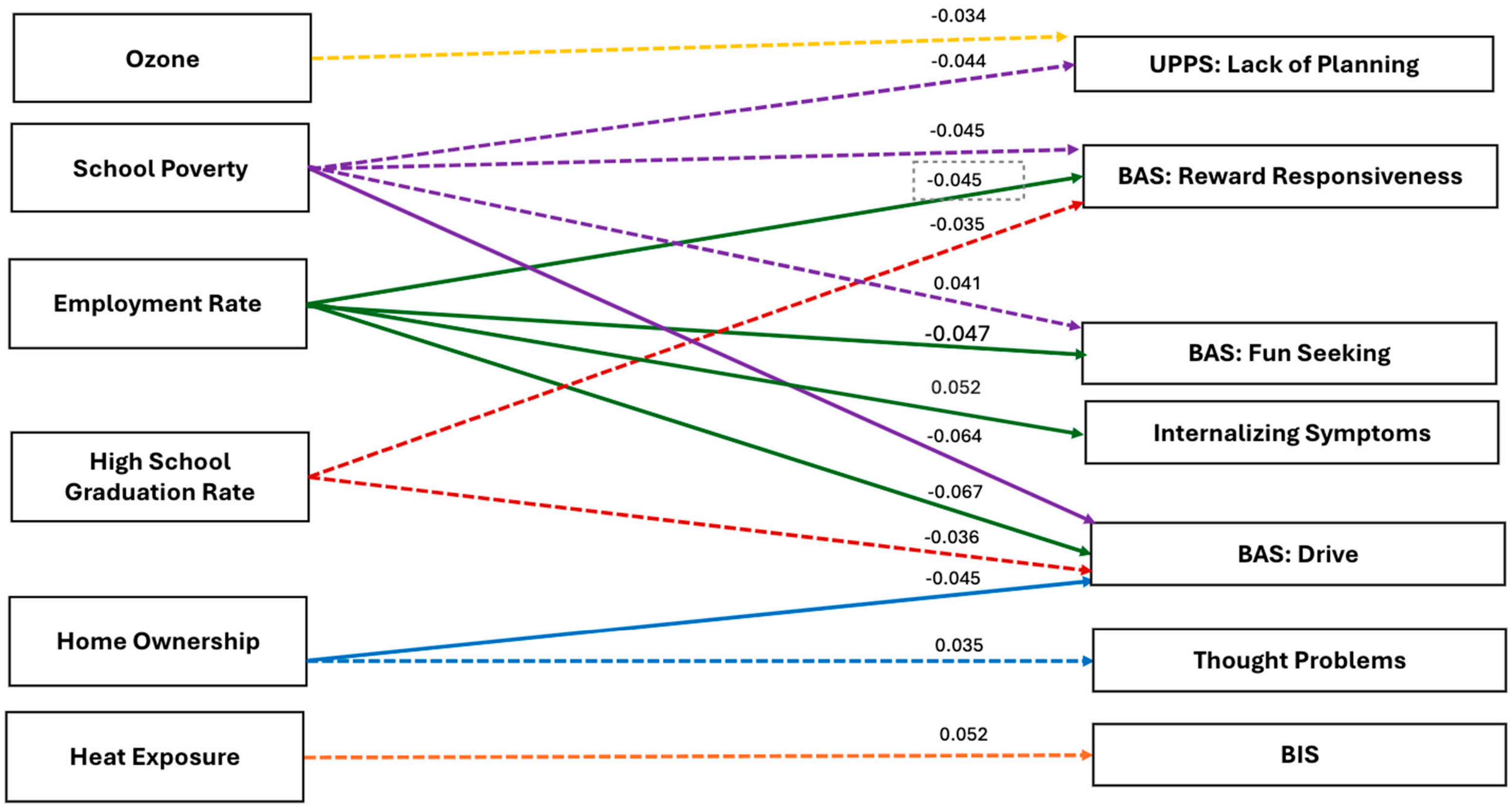

Solid lines indicate FDR adjusted *p* values < 0.01, dotted lines indicate *p* < .05 with the standardized b-value represented.
